# Abilities of preschoolers: comparing different tools

**DOI:** 10.1186/1824-7288-38-3

**Published:** 2012-01-26

**Authors:** Matteo Chiappedi, Erika Maffioletti, Fausta Piazza, Nicole D'Adda, Marta Tamburini, Umberto Balottin

**Affiliations:** 1 Don Carlo Gnocchi ONLUS Foundation, Milan, Italy; 2 Child Neuropsychiatry Unit, IRCCS "C. Mondino" Foundation, University of Pavia, Pavia, Italy

## Abstract

**Background:**

There is a strong need for studies evaluating tests in terms both of psychometric properties (i.e. their efficacy or ability to be helpful in reaching a diagnosis) and of their cost-effectiveness (i.e. their efficiency). These data are essential for planning a correct evaluation to identify children's needs (both educational and abilitative).

**Methods:**

We evaluated 58 children attending for the first time the last year of the Scuola dell'Infanzia. Parental view was obtained with Child Behaviour Check-List and Conners' Rating Scales - Revised, and family socio-economic status was evaluated using Hollingshead's Four Factor Index; teacher compiled the IPDA questionnaire; children were administered Raven's Progressive Matrices, Modified Bell Cancellation Test, BVN 5-11 (a neuropsychological battery).

**Results:**

A correlational analysis was conducted using Spearman's Rho (since variables were not normally distributed). These asymptomatic children show a good global cognitive functioning, but also a deficit of attention and of Executive Functions. Some of the tests used seem more cost-effective than others and there are some redundancies in information obtained.

**Conclusions:**

Our data show that there are significant correlations between different neuropsychological and behavioural measures. It is therefore possible to rationalize diagnostic protocols without a significant information reduction. A deeper analysis will require a preliminary definition of the psychometric properties of used tools.

## Background

Different neuropsychological functions have been shown in pre-schoolers to be more closely correlated to successive school achievement [1]: the integration of several cognitive and perceptual-motor skills is required since the beginning of primary school [2]. English-based literature has focused on letter recognition, spelling ability, phonemic awareness for reading and writing [1,3] and number recognition, quantity processing and counting for mathematical skills [4,5]. Given that Italian, unlike English, has an almost fully transparent orthography, it is perhaps understandable that studies conducted in Italy have shown a preminent role of metaphonological skills [6]. It has been written that these abilities represent a crystallized knowledge, deriving from experiences conducted at home, at the nursery or in other social contests; the role of the so called "g factor" (fluid intelligence, independent from experience) has been stressed especially for higher level and more complex cognitive activities [7].

Executive Functions (EF) have been implied both in relational development in childhood and learning; they can be defined as cognitive processes implied in behavioural regulation and include cognitive flexibility, impulse control, working memory, goal-directed planning and regulation of activity [8]. The role of EFs has been demonstrated for both reading/writing and mathematical skills [9,10],

Among EFs, a special role is probably played by attention, in its different forms and components [11,12].

Learning disabilities are an important risk factor for academic dropout and can influence the social and emotional wellbeing of the child [13]. A learning disability can be cause and/or consequence of an emotional problem, which in turn can compromise academic and relational results [14]. A timely diagnosis of any deficit in skills connected with the possibility to learn is therefore important in order to plan an effective strategy to reduce these deficits and to maximize learning possibilities.

In common neuropsychiatric practice, information is collected from different sources (children themselves, parents, teachers and so on) with different tools (including tests, questionnaires or clinical interviews). In slump time as now, when high costs are a problem and are increasingly reported for Health Systems of Western Societies, we need to be worried about improving not only the efficacy of our tools, but also their efficiency. It would be therefore useful to have data not only about psychometric properties of commonly used tests, but also about cost-effective strategies to use them.

## Methods

Fifty-eight Italian children (25 females, 33 males) attending for the first time the last year of the pre-shool in the "Terzo Circolo" of Pavia were enrolled in this study; their age was between 5 and 6 years. We excluded children with signs of neurological or psychiatric disorders and/or born pre-term. All parents, acting as legal guardians, signed an informed consent and all data were collected and analyzed following the Helsinki declaration.

Children were evaluated in order to obtain information from three main sources, namely parents, teachers and children themselves. To this aim, we used:

1) for parents: Child Behavior CheckList, a questionnaire by Achenbach and co-workers which explores many different domains of functioning [15]; Conners' Rating Scales - Revised, a questionnaire meant to explore perceived behaviours connected with attention deficit and/or hyperactivity [16]; Hollingshead's Four Factor Index, a rather simple tool quantifying the socio-economic status of the family [17];

2) for teachers: IPDA questionnaire, by which the teacher is supposed to express a quantified evaluation of the child's functioning [18];

3) for children: Raven's Progressive Matrices (coloured form), to study learning independent and culture-free intelligence [19]; Modified Bell Cancellation Test, to evaluate attentive skills [20]; BVN 5-11, a battery of neuropsychological tests developed for children aged from 5 to 11 years old [21]. This part of the evaluation was administered during normal school time, inside the school, in a room commonly used for "attention requiring activities".

Descriptive statistics for all variables tested are depicted in Table 1.

**Table 1:** Descriptive statistics for tested variables

Measure	Applied to	Range
Raven's Progressive Matrices	58	6 - 32

Modified Bell Cancellation Test:		

Rapidity	58	6 - 51

Accuracy	58	30 - 129

BVN 5-11		

Auditory discrimination	58	60.4 - 119.7

Non-words repetition	55	87.9 - 122.2

Phonemic analysis	46	76.1 - 131.1

Phonemic fusion	31	90.0 - 177.3

Naming	58	61.9 - 140.6

Syntactic comprehension	57	0 - 136.6

Digit span	58	86.3 - 144.1

Corsi Test	58	68.1 - 132.1

Word pairs learning	53	80.9 - 174.6

Word memory	56	74.7 - 152.7

Short term memory	58	59.8 - 143.5

Long term memory	58	60.4 - 128.9

Praxic verbal skills	56	79.5 - 124.5

Praxic imitative skills	56	68.3 - 123.8

Tower of London	57	68.8 - 144.1

Phonemic fluency	54	86.9 - 147.5

Categorical fluency	58	64.2 - 130.0

Visual discrimination	57	75.0 - 117.2

Visual quantity judgement	57	43.1 - 128.1

Auditory quantity judgement	58	58.9 - 135.3

Auditory attention	56	34.4 - 141.5

Visual attention	55	77.8 - 139.0

Counting	58	40.5 - 98.0

CBCL	56	

CRS-R	55	

IPDA	57	88 - 172

Hollingshead's Four Factor Index	54	12 - 63.5

Measures derived from BVN 5-11 have a range expressed in terms of Standard Score. CBCL and CRS-R do not have a reported range because many indexes can be calculated (see also Table 3). IPDA has a range expressed for the complete score. Hollingshead's Four Factor Index has a range expressed in terms of child's score for Socio-Economic Status.

We also tried to quantify the cost of the tools, taking into account the cost of the material used but also the amount of time needed to present the test.

We analyzed obtained data with MedCalc (TM) and SPSS-PC (TM) version 15 in order to compare these tests and to identify a rational evaluation strategy to be used in asymptomatic children. Given that variables were ordinal but not normally distributed (as resulted from Kolgomorov-Smirnoff test) we used Spearman's Rho to evaluate correlations significance.

## Results

### Subjects studied

Descriptive statistics concerning our patients are depicted in Table 1.

Socio-economic status, described following Hollingshead's recommendations [17], seems to mimic the distribution of the general population.

Most children have a good or excellent cognitive functioning (96.4% scores higher than 50^th ^percentile in Raven's Progressive Matrices). Parents do not seem to identify any particular problem in their children, since mean values in both CBCL and Conners' Rating Scales - Revised are near 50 (i.e. normal, since these tests results are given as T scores); no child fell into the pathological range for any group of symptoms. Taken together, these data seem particularly good even considering that children with known psychiatric and/or neurologic disorders were excluded from our sample.

On the other hand, teachers describe children in a more distributed way, with significant peaks in the highest functioning class (50% of children obtained a score higher than 75^th ^percentile) and in the "frail but not poor group" (38% fell between the 10^th ^and the 25^th ^percentile).

As to BVN 5-11, most of the children performances were in the normal range but some tests of the battery were frequently refused; this is particularly true for Phonemic Fusion, a meta-phonological test which was accepted by only 31 children (53.4%).

Most children showed a poor attentive performance at the Modified Bell Cancellation Test, both in terms of Rapidity (i.e. fixation attention; 73% of children fell below the 25^th ^percentile) and of Accuracy (i.e. sustained attention; 60% of children fell below the 25^th ^percentile).

### Correlation between scores

Statistically significant correlations are given in Table 2. It must be noted that a large number of comparisons were performed; although a stepwise approach was used, we cannot definitely rule out the existence of Type I errors (since this technique is known to be less conservative than, for instance, a Bonferroni correction).

**Table 2:** Significant correlation between tests

Test	Correlates with	Significance	Correlation
Raven's Progressive Matrices	Auditory discrimination	P < 0.001	- 0.492

	Phonemic analysis	P = 0.002	- 0.458

	Corsi test	P = 0.007	- 0.357

	Word pairs learning	P = 0.005	- 0.386

	Auditory attention	P = 0.01	- 0.347

	Tower of London	P < 0.001	- 0.475

	Visual discrimination	P = 0.005	- 0.376

Modified Bell Cancellation Test:			

Rapidity	Corsi test	P = 0.047	- 0.262

	Short term memory	P = 0.001	- 0.432

	Long term memory	P = 0.048	- 0.260

	Auditory attention	P = 0.026	- 0.297

	Tower of London	P = 0.01	- 0.339

	Categorical fluency	P = 0.003	- 0.388

Correctness	Auditory discrimination	P = 0.01	- 0.336

	Corsi test	P = 0.022	- 0.299

	Short term memory	P < 0.001	- 0.454

	Long term memory	P = 0.014	- 0.322

	Auditory attention	P = 0.021	- 0.307

	Tower of London	P = 0.013	- 0.326

	Categorical fluency	P = 0.008	- 0.346

	Phonemic fluency	P = 0.001	- 0.424

	Visual discrimination	P = 0.037	- 0.277

	Social problems (CBCL)	P = 0.028	+ 0.297

Hollingshead's Four Factor Index	Auditory discrimination	P < 0.001	- 0.518

	Phonemic analysis	P < 0.001	- 0.571

	Phonemic fusion	P = 0.02	- 0.414

	Digit span	P = 0.003	- 0.294

	Word pairs learning	P = 0.004	- 0.396

	Visual attention	P = 0.023	- 0.318

	Tower of London	P = 0.001	- 0.432

	Phonemic fluency	P < 0.001	- 0.673

	Categorical fluency	P = 0.004	- 0.385

	Visual discrimination	P = 0.005	- 0.381

	Attention deficit's DSM IV symptoms (CRS-R)	P = 0.04	+ 0.182

	Raven's Progressive Matrices	P = 0.0013	+ 0.340

	IPDA	P < 0.001	+ 0.511

IPDA	Auditory discrimination	P = 0.002	- 0.410

	Phonemic analysis	P = 0.03	- 0.325

	Word pairs learning	P = 0.012	- 0.347

	Long term memory	P = 0.039	- 0.275

	Phonemic fluency	P = 0.002	- 0.403

	Externalizing problems (CBCL)	P = 0.045	+ 0.272

	Total problems (CBCL)	P = 0.034	+ 0.286

	Perfectionism (CBCL)	P = 0.017	+ 0.324

	Psycho-somatic problems (CBCL)	P = 0.012	- 0.341

	SES	P < 0.001	+ 0.511

As to Raven's Progressive Matrices, all correlations are positive (i.e. better results in other tests tends to predict a better result in Raven's Progressive Matrices).

The same applies for the Modified Bell Cancellation Test; in this case, however, there are differences between Rapidity and Accuracy in terms of correlated tests.

Hollingshead's Four Factor Index correlates with many tests and subtests, again with a positive trend (i.e. a higher Socio-Economic Status predicts better results but also a higher level of parental perception of child's problems, particularly in terms of attention deficit).

The IPDA correlates with many other tests and subtests, among which the positive correlation with the Socio-Economic Status and the negative one with externalizing and general problems signalled by parents (CBCL scores) are particularly noteworthy (i.e. more problems correlate with a teacher's perception of reduced child's skills).

CBCL's and CRS-R's correlations are reported in Table 3. These two questionnaires correlate to each other in a positive way (i.e. children with higher reported problems in one questionnaire tend to have higher reported problems in the other questionnaire).

**Table 3:** Significant correlations between CBCL and CRS-R

CBCL score	Correlates with CRS score	Correlation	Significance
Total	Oppositivity	0.639	P < 0.001

	Cognitive problems	0.510	P < 0.001

	Hyperactivity	0.543	P < 0.001

	Anxiety/Shyness	0.647	P < 0.001

	Perfectionism	0.366	P = 0.006

	Psychosomatic problems	0.274	P = 0.043

	ADHD symptoms	0.578	P < 0.001

	Fidgety/Impulsivity	0.667	P < 0.001

	Emotional instability	0.414	P = 0.002

	Clinical Global Impression	0.662	P < 0.001

	Attention deficit (DSM IV)	0.626	P < 0.001

	Hyperactivity (DSM IV)	0.524	P < 0.001

	ADHD symptoms in DSM IV	0.629	P < 0.001

Internalizing problems	Oppositivity	0.585	P < 0.001

	Cognitive problems	0.355	P = 0.008

	Hyperactivity	0.447	P = 0.001

	Anxiety/Shyness	0.569	P < 0.001

	Perfectionism	0.312	P = 0.02

	Psychosomatic problems	0.390	P = 0.003

	ADHD symptoms	0.390	P = 0.003

	Fidgety/Impulsivity	0.445	P = 0.001

	Emotional instability	0.402	P = 0.002

	Clinical Global Impression	0.461	P < 0.001

	Attention deficit (DSM IV)	0.432	P = 0.001

	Hyperactivity (DSM IV)	0.384	P = 0.004

	ADHD symptoms in DSM IV	0.404	P = 0.002

Externalizing problems	Oppositivity	0.567	P < 0.001

	Cognitive problems	0.472	P < 0.001

	Hyperactivity	0.505	P < 0.001

	Anxiety/Shyness	0.433	P = 0.001

	Perfectionism	0.407	P = 0.002

	ADHD symptoms	0.624	P < 0.001

	Fidgety/Impulsivity	0.718	P < 0.001

	Emotional instability	0.354	P = 0.008

	Clinical Global Impression	0.700	P < 0.001

	Attention deficit (DSM IV)	0.560	P < 0.001

	Hyperactivity (DSM IV)	0.515	P < 0.001

	ADHD symptoms in DSM IV	0.608	P < 0.001

### Cost evaluation of different tools

Table 4 presents the economic evaluation of all tests used in this study.

**Table 4:** Cost of tests used

TEST	Base kit cost	Materials cost	Time needed	Operator cost	Full cost
BVN 5-11	169	5	180	90	95

Raven's Progressive Matrices	275	3	40	20	23

Modified Bell Cancellation Test	0	1	20	10	11

CBCL	248	9	20	10	19

Conners' Rating Scales - Revised	263	11	15	7,5	18,5

IPDA	29	3	20	10	13

Hollingshead's Four Factor Index	0	0	10	5	5

We present the cost of the basic kit; the cost of materials for a single administration (approximated to the whole euro), described as one hundredth of the cost of the basic kit plus the cost of any consumable material necessary; the time needed for a single administration (given in minutes and derived from the manual of the test but also from our experience with children in this study); the cost of the operator (administration and scoring), assuming a standardized full cost of 30 Euro per hour; the full cost of a single administration (resulting from the cost of the materials and the cost of the operator).

## Discussion

Learning can be defined as a complex process, involving motivation, emotions, memory and other cognitive processes that are necessary to acquire meaningful information useful in reaching one or more specific goals. The assessment of learning possitibilities in a child should therefore include many different aspects, ranging from an evaluation of motivation and emotional balance to a wide range of cognitive skills [22].

Our study offers data concerning the correlations existing between different evaluation tools and analyzes the economical aspects of their use.

To start with, it should be stressed that the existence of a statistically significant correlation does not imply a cause - effect connection. In our study, correlations between some measures used were found.

Moreover, for most of the tests used (as well as for most tests used in child neuropsychiatry in general) psychometric properties are poorly defined; this constitutes a major limitation for this study but also, more importantly, a relevant problem for everyday clinical practice.

Teachers description of the child, quantified by the IPDA questionnaire, is correlated to child's ability to use language and to manipulate its parts (so called "metaphonological skills"). This was probably to be expected, given that teachers are supposed to be interested in the cognitive functioning and to exploit cognitive and linguistic skills to obtain learning. On the other hand, the reason why their views correlate with the Socio-Economic Status of the family and with parental perception of behavioural problems (especially externalizing problems) is not self-evident. Children from poorer families and/or whose parents report more externalizing problems (i.e. a tendency towards provocative or disruptive or hyperactive behaviours) tend to be seen as "less able" from their teachers, even when they have adequate neuropsychological skills; the interaction between these factors is not completely clear from our data.

One could speculate that externalizing behaviours could be "disturbing" for the learning process, but also for the teacher herself. This view, however, does not explain the SES factor.

Raven's Progressive Matrices are supposed to allow the investigation of intelligence in a culture-free and learning independent way. It is interesting to note that the score obtained by the child is significantly correlated to his attentive and visual processing skills: this could have been predicted from an analysis of the proposed test, which is based on the ability to perform a visual scanning of the matrices and to concentrate on the task. The correlation with Executive Functions and therefore with the ability to face complex and new situations in a successful way has been reported for different intelligence tests [23]. It is worth noting, as to this point, that Phonemic Analysis and Phonemic Fusion, which are considered subtests exploring the metaphonological correlates of Executive Functions, were performed by 46 (79.3%) and 31 (53.5%) of children only: this may imply that these children, although tested in a well-known setting and with a known adult to assist them, were unable even just to try to answer to this unfamiliar request.

Socio-Economic Status is known to influence cognitive development and a variety of cognitive abilities [24] and our data seem to confirm previous and widely reported findings. It should be noted, however, that most published reports have been obtained in children from English-speaking countries. Our data, confirming previous findings, stand with the hypothesis that considers SES as a relevant factor in determining one's cognitive development.

CBCL and Conners' Rating Scales - Revised seem to be rather similar in providing information about parental view. This finding is not new, as previous research with children with ADHD has shown that the inclusion of multiple parent questionnaires does not lead to incremental validity [25]. Our study adds to the demonstration that these questionnaires are widely correlated also in a non-clinical population.

It must be stressed that CBCL and CRS-R profile depend on the real functioning of the child but also on the interpretation of this functioning given by parents; this might explain why parents seemed not to consider their children affected by an attention deficit although most of them performed poorly in an attention test (Modified Bell Cancellation Test). This attention test has published normative data for this age class [20]: this suggests that most children show a real difficulty in the attentive field and is consistent with the difficulties we found for Visual and Auditory Attention as measured in the BVN 5-11.

It is also worth noting that the perception of the child problems tended to increase in families with a higher Socio-Economic Status, showing that the score is probably influenced by parent's view of how a child should behave.

BVN 5-11 is the only tool in our research protocol which allows to reach a wide functional profile of the child, which can be predicted only partially through the other tools used. This can be useful to identify specific deficits and to plan an adequate intervention, both in terms of an adequate kindergarten activity and/or of an abilitative treatment.

Taking costs into account (see Table 4), Raven Progressive Matrices seem to be not only valuable (as they quantify cognitive functioning) but also cost-effective.

CBCL and Conners' Rating Scales - Revised seem to overlap in terms of diagnostic utility and cost: in order to reduce unnecessary expenses, it could be enough to propose one of these questionnaires (which also require a rather long time to be filled by parents, a fact which could lead to inaccuracies [26]).

IPDA questionnaire is highly economical, but our data show a correlation of its score with parameters which are not directly related to the child, such as the Socio-Economic Status of the family. Therefore, even if our study doesn't demonstrate any causal relation, it should be used cautiously.

The evaluation of the Socio-Economic Status, using a tool such as Hollingshead's Four Factor Index, is both cost-effective and interesting, given the correlations with many aspects of cognitive functioning and behaviour (evidenced both in scientific literature and in our data).

The Modified Bell Cancellation Test seems both low-cost and useful, but it is possible that its role is over-estimated because the large majority of children enrolled had a poor attentive performance.

To end with, BVN 5-11 proved to be rather expensive, but is the only test included in our research protocol which allows us to obtain a sufficiently complete neuropsychological profile of the child, which is fundamental for planning a correct treatment strategy. It could be discussed, however, if this is the neuropsychological battery of choice or if others should be preferred.

## Conclusions

A major and often claimed problem of the Italian Health System is the lack of resources. It is therefore important to choose evaluation tools on the basis of their ability to help understand children and their functional and diagnostic profile (efficacy) but also on their cost-effectiveness (efficiency).

Our study seems to offer valuable information not only on a non-clinical population of pre-schoolers, but also on the possibility of choosing a cost-effective evaluation protocol.

It must be said however that, although these tests are widely used in clinical practice, there is room for many improvements in terms of their cost-effectiveness but also of their psychometric properties, which are in general poorly defined. This represents a major problem not only for research but also, and most importantly, for clinical practice.

The major limitation of our study is that our data do not allow us to draw any conclusion on these psychometric properties, and therefore on the efficacy, of the tests used. This also limits our possibility to deepen the economical analysis, because a basic factor (i.e. test efficacy) is not completely defined.

## Competing interests

The authors declare that they have no competing interests.

## Authors' contributions

MC, EM, FP and UB planned the study. MC, EM, FP, ND and MT conducted children's evaluation. MC and UB conducted statistical analysis. All authors contributed to draft the manuscript, which they read and approved in the final version.
